# Recommendations on how to provide cardiac rehabilitation services during the COVID-19 pandemic

**DOI:** 10.1007/s12471-020-01474-2

**Published:** 2020-07-16

**Authors:** H. M. C. Kemps, R. W. M. Brouwers, M. J. Cramer, H. T. Jorstad, E. P. de Kluiver, R. A. Kraaijenhagen, P. M. J. C. Kuijpers, M. R. van der Linde, E. de Melker, S. F. Rodrigo, R. F. Spee, M. Sunamura, T. Vromen, M. E. Wittekoek, Committee for Cardiovascular Prevention and Cardiac Rehabilitation of the Netherlands Society of Cardiology, H. M. C. Kemps, R. W. M. Brouwers, M. J. Cramer, H. T. Jorstad, E. P. de Kluiver, R. A. Kraaijenhagen, P. M. J. C. Kuijpers, M. R. van der Linde, E. de Melker, S. F. Rodrigo, R. F. Spee, M. Sunamura, T. Vromen, M. E. Wittekoek

**Affiliations:** 1 Department of Cardiology, Máxima Medical Centre, Veldhoven, The Netherlands; 2 Department of Industrial Design, Eindhoven University of Technology, Eindhoven, The Netherlands; 3 Department of Cardiology, University Medical Centre, Utrecht, The Netherlands; 4 Department of Cardiology, Amsterdam University Medical Centre, University of Amsterdam, Amsterdam, The Netherlands; 5 Isala Heart Centre, Zwolle, The Netherlands; 6 CardioVitaal Cardiac Rehabilitation, Amsterdam, The Netherlands; 7 Department of Cardiology, Maastricht University Medical Centre, Maastricht, The Netherlands; 8 Department of Cardiology, Nij Smellinghe Hospital, Drachten, The Netherlands; 9 Department of Cardiology, OLVG, Amsterdam, The Netherlands; 10 Basalt Rehabilitation, Leiden, The Netherlands; 11 Capri Cardiac Rehabilitation, Rotterdam, The Netherlands; 12 HeartLife Klinieken, Utrecht, The Netherlands

**Keywords:** Cardiac rehabilitation, Cardiac telerehabilitation, Telemonitoring, Coronary artery disease

## Abstract

The ongoing coronavirus disease 2019 (COVID-19) crisis is having a large impact on acute and chronic cardiac care. Due to public health measures and the reorganisation of outpatient cardiac care, traditional centre-based cardiac rehabilitation is currently almost impossible. In addition, public health measures are having a potentially negative impact on lifestyle behaviour and general well-being. Therefore, the Working Group of Cardiovascular Prevention and Rehabilitation of the Dutch Society of Cardiology has formulated practical recommendations for the provision of cardiac rehabilitation during the COVID-19 pandemic, by using telerehabilitation programmes without face-to-face contact based on current guidelines supplemented with new insights and experiences.

## Introduction

During the coronavirus disease 2019 (COVID-19) pandemic many elective and outpatient healthcare activities have been cancelled or postponed. Also, a decline in admissions due to cardiac disease has been observed [1]. In many Dutch hospitals and specialised rehabilitation clinics, cardiac rehabilitation (CR) programmes have been shut down partially or even completely. In particular, group-based exercise training interventions and exercise testing are hardly possible. In addition, the public health measures implemented during the COVID-19 pandemic, above all quarantine and isolation, have been shown to be associated with anxiety, anger and stress, which is associated with unhealthy lifestyle behaviour, including a reduction in physical activity and an unhealthy diet [2]. Therefore, there is an urgent need for upscaling and reorganisation of CR and secondary prevention services. Recently, the Working Group of Cardiovascular Prevention and Rehabilitation of the Dutch Society of Cardiology has formulated practical recommendations for CR during the COVID-19 pandemic. These recommendations are partly based on a recent statement from the Secondary Prevention and Rehabilitation Section of the European Association of Preventive Cardiology [3], supplemented with insights from the recently published Dutch Telerehabilitation guidelines [4] and practical experiences from the largest Dutch CR centres. The importance of continuing the delivery of (cardiac) rehabilitation during the COVID-19 pandemic by means of telerehabilitation programmes without face-to-face contact is emphasised by the prompt reimbursement of these programmes by the Netherlands Health Institute (NZa) and with support from the Netherlands Health Institute (ZiN).

## General recommendations

- Support acute cardiac wards in providing summarised but highlighted important information/recommendations on secondary prevention (not forgetting physical activity and mental impact) before hospital discharge.
- In the case of shortened CR programmes, concentrate efforts on the main core components (i.e. lifestyle risk management, psychosocial support, medical advice, education) with an individualised approach based on psychological symptoms, residual cardiac risk and lifestyle assessment.
- Replace face-to-face sessions by remote assessment and monitoring/guiding, according to local equipment and expertise (telephone, text messaging, e‑mails, video consultations, web-based platforms and applications).
- Perform patient assessment and risk stratification with an exercise test whenever possible. If not possible, use other tools to assess the cardiovascular risk and physical fitness in order to provide personalised exercise advice and to guide telerehabilitation (see Tab. 1).
- For COVID-19-positive patients, postpone the exercise programme if fever, symptoms or other signs of COVID-19 infection are present [3]. Evaluate exercise resumption on an individual basis. In general, in patients with light-to-moderate symptoms, gradually restart the exercise programme after a fever-free period of 1 week and a symptom-free period of 48 h. Whenever possible, do not postpone all other CR components but provide them remotely (see Tab. 1).
- For general safety and hygiene measures take into account the recommendations of the Dutch National Institute for Public Health and the Environment (*Rijksinstituut voor Volksgezondheid en Milieu*,RIVM) and the Dutch Federation of Medical Specialists (*Federatie Medisch Specialisten*, FMS) [5, 6].

**Table 1:** Modes of delivery of cardiac rehabilitation (*CR*) according to current guidelines and recommendations for alternatives during the coronavirus disease 2019 (*COVID-19*) pandemic

	Current guideline [7]	Recommendations during COVID-19 pandemic
Assessment	Assessment of five domains according to the Dutch clinical algorithms for cardiac rehabilitation (*Poliklinische Indicatiestelling Hartrevalidatie*) 2012 [8]: physical functioning, psychological functioning, social functioning, cardiovascular risk profile, risk behaviour	Assessment of five domains according to the Dutch clinical algorithms for cardiac rehabilitation (*Poliklinische Indicatiestelling Hartrevalidatie*) 2012 [8]: If exercise testing is not possible use alternative validated tools to assess physical fitness for risk asessment (see ‘Exercise programme’), e.g.: – HUNT non-exercise prediction model [9] – The 2‑min step test [10] or other validated submaximal exercise tests may be considered if performed under direct video-supervision
Exercise programme	Group sessions at CR centre Telerehabilitation according to the Dutch Society of Cardiology (NVVC) guideline addendum concerning telerehabilitation [4]: training prescription and risk assessment based on ergometry and monitoring with heart rate sensor and activity tracker	If a recent exercise test is available, telerehabilitation should be performed according to the Dutch Society of Cardiology (NVVC) guideline addendum concerning telerehabilitation [4]. When no exercise test is available, telerehabilitation can be applied in an adapted form: 1a. For non-complex patients according to the Dutch Society of Cardiology Practice Guidelines for Cardiac Rehabilitation (*NVVC praktijkrichtlijn Hartrevalidatie*) [11] and individual judgement by the cardiologist: – Use a validated tool to assess physical fitness. – Direct supervision using a secure video connection – Monitoring of symptoms, blood pressure and heart rate before and after sessions – Groups are composed according to fitness levels, determined by a validated assessment tool (see ‘Assessment’). – Aerobic training intensity is monitored after each session using the Borg scale, aiming at a score of 12–14 [12]. – During sessions the physical therapist should be accompanied by a physician or specialised nurse/physician assistant who is enabled to communicate with individual patients separately when necessary. – During sessions, the patient should be accompanied by someone who can contact/be contacted if patients develop symptoms of dypnoea, chest pain dizziness or palpitations. – Training sessions should be complemented with educational material on exercise (digital or on paper). 1b. For complex patients according to the Dutch Society of Cardiology Practice Guidelines for Cardiac Rehabilitation (*NVVC praktijkrichtlijn Hartrevalidatie*) when no exercise test is available: – Provide individual advice and coaching by physical therapist/exercise specialist or sports physician. – Focus exercise prescription on low-to-moderate intensity aerobic exercise (Borg scale ≤12) at the level of activities of daily living (flexibility, coordination and muscle strength). – Initial session(s) under direct supervision using a secure video connection with monitoring of symptoms, blood pressure and heart rate before and after sessions. – During these sessions the patient should be accompanied by someone who can contact/be contacted if patients develop symptoms of dypnoea, chest pain dizziness or palpitations. – Training sessions should be complemented by educational material on exercise (digital or on paper). 2. When both exercise testing and telerehabilitation are unavailable: Individual advice and coaching by physical therapist/exercise specialist or sports physician based on the individuals’ rehabilitation goals, focusing on unsupervised aerobic low-to-moderate intensity exercise (Borg scale ≤12). The advice should be complemented by educational material (digital or on paper) and patients should be contacted on a regular basis by telephone
Psychoeducational prevention (PEP) programme	Group sessions at CR centre Remote PEP programme according to NVVC guideline: individual intake and remote guidance using an online platform	Individual intake and group sessions using a secure group video connection Remote PEP programme according to NVVC guideline: individual intake and remote guidance using an online platform
Education programme	Group sessions at CR centre	Individual or group remote educational consultation(s) Online material and/or interactive e‑learning
Relaxation programme	Group sessions at CR centre	Group or individual sessions using a secure video connection
Individual treatment dietician/psychologist/social worker	Outpatient consultations	Remote consultations using a secure video connection or telephone
Individual medical treatment	Outpatient consultations Lab testing and physical exam, blood pressure	Remote consultations using a secure video connection or telephone Lab testing, blood pressure and heart rate measurement at home using validated sensors and educational material

Specific recommendations are presented in Tab. 1.
